# Progress Toward Measles Elimination — European Region, 2009–2018

**DOI:** 10.15585/mmwr.mm6817a4

**Published:** 2019-05-03

**Authors:** Laura A. Zimmerman, Mark Muscat, Simarjit Singh, Myriam Ben Mamou, Dragan Jankovic, Siddhartha Datta, James P. Alexander, James L. Goodson, Patrick O’Connor

**Affiliations:** ^1^Global Immunization Division, Center for Global Health, CDC; ^2^Vaccine Preventable Diseases and Immunization, European Regional Office, World Health Organization, Copenhagen, Denmark.

In 2010, all 53 countries* in the World Health Organization (WHO) European Region (EUR) reconfirmed their commitment to eliminating measles and rubella and congenital rubella syndrome (*1*); this goal was included as a priority in the European Vaccine Action Plan 2015–2020 (*2*). The WHO-recommended elimination strategies in EUR include 1) achieving and maintaining ≥95% coverage with 2 doses of measles-containing vaccine (MCV) through routine immunization services; 2) providing measles and rubella vaccination opportunities, including supplementary immunization activities (SIAs), to populations susceptible to measles or rubella; 3) strengthening surveillance by conducting case investigations and confirming suspected cases and outbreaks with laboratory results; and 4) improving the availability and use of evidence for the benefits and risks associated with vaccination (*3*). This report updates a previous report (*4*) and describes progress toward measles elimination in EUR during 2009–2018. During 2009–2017, estimated regional coverage with the first MCV dose (MCV1) was 93%–95%, and coverage with the second dose (MCV2) increased from 73% to 90%. In 2017, 30 (57%) countries achieved ≥95% MCV1 coverage, and 15 (28%) achieved ≥95% coverage with both doses. During 2009–2018, >16 million persons were vaccinated during SIAs in 13 (24%) countries. Measles incidence declined to 5.8 per 1 million population in 2016, but increased to 89.5 in 2018, because of large outbreaks in several EUR countries. To achieve measles elimination in EUR, measures are needed to strengthen immunization programs by ensuring ≥95% 2-dose MCV coverage in every district of each country, offering supplemental measles vaccination to susceptible adults, maintaining high-quality surveillance for rapid case detection and confirmation, and ensuring effective outbreak preparedness and response.

## Immunization Activities

Since 2002, all 53 countries in EUR have included 2 MCV doses in routine childhood vaccination schedules. WHO and the United Nations Children’s Fund (UNICEF) estimate vaccination coverage for all countries in the region using annual, government-reported administrative coverage data (calculated as the number of doses administered divided by the estimated target population) and vaccination coverage surveys (*5*). During 2009–2017, annual estimates of MCV1 coverage were available for all 53 countries, and the number of countries with annual MCV2 coverage estimates increased from 47 (89%) to 52 (98%). During 2009–2017, regional coverage estimates for MCV1 and MCV2 ranged from 93% to 95% and 73% to 90%, respectively (Figure). In 2017, 30 (57%) countries achieved ≥95% MCV1 coverage, and 15 (28%) had ≥95% estimated coverage with both doses (Table 1). During 2009–2017, >16 million persons were vaccinated in 21 SIAs conducted in 13 countries (Supplementary Table, https://stacks.cdc.gov/view/cdc/77666). Reported administrative vaccination coverage was ≥95% in nine (43%) SIAs, and the weighted average SIA coverage was 88%; no post-SIA coverage surveys were reported.

**FIGURE:**
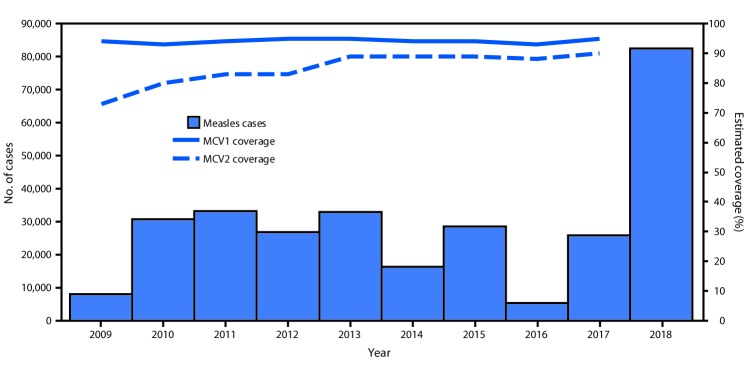
Estimated coverage with the first and second doses of measles-containing vaccine* and the number of confirmed measles cases**^†^** — World Health Organization (WHO) European Region, 2009–2018**^§^** **Abbreviations:** MCV1 = first dose of a measles-containing vaccine; MCV2 = second dose of a measles-containing vaccine. * WHO and United Nations Children’s Fund estimates, July 15, 2018, update. https://www.who.int/immunization/monitoring_surveillance/data/en/. ^†^ Cases reported to WHO, as of March 1, 2019. https://www.who.int/immunization/monitoring_surveillance/data/en/. ^§^ Date range for estimated coverage = 2009–2017; date range for confirmed measles cases = 2009–2018.

**TABLE 1:** Measles-containing vaccine (MCV) schedule, estimated coverage with the first and second doses of MCV,* number of confirmed measles cases,^†^ and confirmed measles incidence, by country — World Health Organization (WHO) European Region, 2009, 2017, and 2018

Country	MCV schedule^§^		2009				2017				2018**	
			Coverage (%)		No. of measles cases	Measles incidence^¶^	Coverage (%)		No. of measles cases	Measles incidence^¶^	No. of measles cases	Measles incidence^¶^
	Age for MCV1	Age for MCV2	MCV1	MCV2			MCV1	MCV2				
Albania	12 mos	5 yrs	97	98	0	0.0	96	98	12	4.1	1,466	499.6
Andorra	12 mos	3 yrs	98	82	0	0.0	99	94	0	0.0	0	0.0
Armenia	12 mos	6 yrs^††^	96	96	1	0.3	96	97	1	0.3	19	6.5
Austria	10 mos	11 mos	76	64	47	5.6	96	84	94	10.8	77	8.8
Azerbaijan	12 mos	6 yrs	85	83	0	0.0	98	97	0	0.0	71	7.2
Belarus	12 mos	6 yrs	99	99	1	0.1	97	98	1	0.1	235	24.9
Belgium	12 mos	11–12 yrs	95	83	33	3.0	96	85	367	32.1	120	10.4
Bosnia and Herzegovina	12 mos	6 yrs	93	88	0	0.0	69	80	27	7.7	89	25.4
Bulgaria	13 mos	12 yrs	96	93	2,545	341.3	94	92	165	23.3	13	1.8
Croatia	12 mos	6 yrs	95	98	2	0.5	89	95	7	1.7	23	5.5
Cyprus	12–15 mos	4–6 yrs	87	88	0	0.0	90	88	4	3.4	14	11.8
Czech Republic	15 mos	5 yrs	98	98	5	0.5	97	90	149	14.0	199	18.7
Denmark	15 mos	4 yrs	84	85	8	1.4	97	88	4	0.7	8	1.4
Estonia	12 mos	13 yrs	95	96	0	0.0	93	91	1	0.8	10	7.7
Finland	12–18 mos	6 yrs	98	NR	3	0.6	94	92	10	1.8	15	2.7
France	12 mos	18 mos	89	NR	1,541	24.6	90	80	518	8.0	2,913	44.7
Georgia	12 mos	5 yrs	83	71	23	5.4	95	90	96	24.5	2,203	563.8
Germany	11–14 mos	15–23 mos	97	93	572	7.1	97	93	936	11.4	532	6.5
Greece	12–15 mos	4–6 yrs	99	77	2	0.2	97	83	1,067	95.6	2,193	196.8
Hungary	15 mos	11 yrs	99	99	1	0.1	99	99	36	3.7	14	1.4
Iceland	18 mos	12 yrs	92	93	0	0.0	92	95	3	9.0	0	0.0
Ireland	12 mos	4–5 yrs	90	NR	197	43.1	92	NR	25	5.3	90	18.7
Israel	12 mos	6 yrs	97	92	5	0.7	98	96	16	1.9	2,919	345.3
Italy	13–15 mos	5–6 yrs	90	NR	173	2.9	92	86	5,393	90.9	2,517	42.5
Kazakhstan	12 mos	6 yrs	99	99	0	0.0	99	99	2	0.1	576	31.3
Kyrgyzstan	12 mos	6 yrs	99	98	0	0.0	95	96	5	0.8	1,008	164.4
Latvia	12–15 mos	7 yrs	92	92	0	0.0	96	89	5	2.6	20	10.4
Lithuania	15–16 mos	6–7 yrs	96	94	0	0.0	94	92	2	0.7	30	10.4
Luxembourg	12 mos	15–23 mos	96	NR	0	0.0	99	86	4	6.9	4	6.8
Malta	13 mos	3 yrs	82	85	1	2.4	91	83	0	0.0	5	11.6
Monaco	12 mos	16 mos	92	NR	0	0.0	87	79	0	0.0	0	0.0
Montenegro	13 mos	6 yrs	86	96	0	0.0	58	83	0	0.0	203	322.6
Netherlands	14 mos	9 yrs	96	93	15	0.9	93	90	16	0.9	24	1.4
North Macedonia	12 mos	6 yrs	96	97	3	1.4	83	97	20	9.6	64	30.7
Norway	15 mos	11 yrs	93	96	2	0.4	96	91	1	0.2	12	2.2
Poland	13–15 mos	10 yrs	98	95	162	4.2	96	93	63	1.7	335	8.8
Portugal	12 mos	5 yrs	95	95	3	0.3	98	95	34	3.3	171	16.6
Republic of Moldova^§§^	12 mos	7 yrs	90	98	0	0.0	93	92	0	0.0	340	84.1
Romania	12 mos	5 yrs	96	94	8	0.4	86	75	9,072	461.0	1,087	55.5
Russia	12 mos	6 yrs^¶¶^	98	97	101	0.7	98	97	897	6.2	2,256	15.7
San Marino	15 mos	10 yrs	88	92	0	0.0	82	78	0	0.0	0	0.0
Serbia	12 mos	7 yrs	95	87	0	0.0	86	91	702	79.9	5,076	579.3
Slovakia	14 mos	10 yrs	99	99	0	0.0	96	97	10	1.8	572	105.0
Slovenia	12 mos	5 yrs	95	98	0	0.0	93	94	8	3.8	9	4.3
Spain	12 mos	3–4 yrs	98	90	43	0.9	96	93	157	3.4	225	4.8
Sweden	18 mos	6–8 yrs	97	95	3	0.3	97	95	46	4.6	38	3.8
Switzerland	12 mos	15–24 mos	92	83	999	129.1	95	89	105	12.4	51	6.0
Tajikistan	12 mos	6 yrs	89	93	177	23.7	98	98	651	73.0	0	0.0
Turkey	12 mos	6 yrs	97	88	8	0.1	96	86	69	0.9	557	6.8
Turkmenistan	12–15 mos	6 yrs	99	99	0	0.0	99	99	0	0.0	0	0.0
Ukraine	12 mos	6 yrs	75	68	24	0.5	86	84	4,782	108.1	53,218	1,209.2
United Kingdom	12 mos	40 mos	86	79	1,176	18.7	92	88	280	4.2	953	14.3
Uzbekistan	12 mos	6 yrs	95	8	0	0.0	99	99	0	0.0	22	0.7
**European Region**	**—**	**—**	**94**	**73**	**7,884**	**8.8**	**95**	**90**	**25,863**	**28.1**	**82,596**	**89.5**

**Abbreviations:** MCV1 = first dose of MCV; MCV2 = second dose of MCV; NR = not reported (country did not report coverage for the year specified).

* WHO and United Nations Children’s Fund estimates of national immunization coverage, 2018. https://www.who.int/immunization/monitoring_surveillance/data/en/.

^†^ Includes confirmed cases by laboratory or epidemiologic linkage and clinically compatible cases meeting the WHO clinical case definition of measles for which no adequate specimen was collected and that cannot be epidemiologically linked to a laboratory-confirmed case of measles.

^§^ MCV schedule is the 2017 schedule.

^¶^ Per 1 million population.

** 2018 MCV1 and MCV2 coverage estimates not available.

^††^ Also recommended for males aged 16–17 years who have not previously received 2 MCV doses.

^§§^ Catch-up vaccination at age 15 years is also performed.

^¶¶^ Catch-up monovalent measles vaccine is also recommended for persons aged 18–55 years.

## Surveillance Activities

Measles surveillance data are reported monthly to WHO from all EUR countries either directly or via the European Centre for Disease Prevention and Control.^†^ As of 2018, 47 (89%) countries report case-based measles surveillance data; six (11%)^§^ report aggregate data. Suspected measles cases are investigated and classified as laboratory-confirmed, epidemiologically linked (to a laboratory-confirmed case), clinically compatible, or discarded (a suspected case that does not meet the clinical or laboratory definition) (*6*). The WHO European Measles and Rubella Laboratory Network provides laboratory confirmation and genotyping of measles virus isolates from patients with reported cases (*7*). Key measles case-based surveillance performance indicators include 1) the number of suspected cases discarded as nonmeasles or nonrubella (target: ≥2 per 100,000 population); 2) the percentage of case investigations conducted within 48 hours of report (target: ≥80%); 3) the percentage of suspected cases (excluding those that are epidemiologically linked) with an adequate specimen collected within 28 days of rash onset and tested in a WHO-accredited or proficient laboratory (target: ≥80%); and 4) the percentage of cases for which the origin of infection (i.e., the source of the virus) is determined (target: ≥80%). During 2009–2018, the number of EUR countries that met the target for suspected cases discarded as nonmeasles at the national level increased from one (3%) in 2009 to 10 (21%) in 2018 (Table 2). From 2009 to 2018, the number of countries achieving the targets for timely investigations of suspected cases and adequate specimen collection increased from one (3%) to 24 (51%) and from 13 (36%) to 38 (81%), respectively.

**TABLE 2:** Percentage of countries reporting case-based surveillance (CBS) data monthly that meet surveillance indicator performance targets — World Health Organization (WHO) European Region, 2009–2018

CBS characteristic	Year									
	2009	2010	2011	2012	2013	2014	2015	2016	2017	2018
**No. (%) of countries reporting CBS data monthly**	36 (68)	38 (72)	41 (77)	41 (77)	46 (87)	46 (87)	46 (87)	46 (87)	47 (89)	47 (89)
**% Countries meeting performance targets/surveillance indicator (performance target)**										
Completeness* (≥80%)	75	71	76	90	93	91	24	87	98	100
Timeliness^†^ (≥80%)	31	26	49	85	87	76	11	72	70	79
Discarded cases^§^ (≥2 per 100,000 population)	3	3	2	0	11	7	7	7	13	21
Timely investigation^¶^ (≥80%)	3	5	24	34	33	30	28	26	40	51
Laboratory investigation** (≥80%)	36	50	68	66	61	70	59	61	81	81
Origin of infection^††^ (≥80%)	47	45	41	49	54	48	41	37	62	60

* Percentage of measles or rubella routine surveillance reports submitted from subnational to national level.

^†^ Percentage of measles or rubella routine surveillance reports submitted from subnational to national level by the deadline set by national program.

^§^ The rate of suspected measles or rubella cases investigated and discarded as nonmeasles and nonrubella, using laboratory testing in a proficient laboratory or epidemiological linkage to another confirmed disease.

^¶^ Percentage of suspected measles or rubella cases with an adequate case investigation initiated within 48 hours of case notification.

** Percentage of suspected measles or rubella cases with an adequate specimen collected and tested in a WHO-accredited or proficient laboratory.

^††^ Percentage of confirmed measles or rubella cases for which the origin of infection (i.e., source of virus) has been identified.

## Measles Incidence and Genotypes

During 2009–2018, annual regional measles incidence varied from 8.8 per 1 million population (7,884 cases) in 2009 to an average of 30.1 (average 28,021 cases) during 2010–2015. Incidence declined to a low of 5.8 (5,273 cases) in 2016, before increasing approximately fourteenfold to a high of 89.5 (82,596 cases) in 2018 (Table 1) (Figure). These 82,596 cases were reported from 47 (89%) EUR countries; 73,295 (89%) were reported by eight countries: Ukraine (53,218 cases; 64% of total); Serbia (5,076; 6%); France (2,913; 4%); Israel (2,919; 4%); Georgia (2,203; 3%); Greece (2,193; 3%); Italy (2,517; 3%); and Russia (2,256; 3%). The highest measles incidences in 2018 were in Ukraine (1,209.2 per 1 million) and Serbia (579.3). Among all measles cases reported in 2018, adults aged ≥20 years accounted for 30,561 (37%). The countries with the highest proportions of adult measles cases were Italy (68%), Serbia (67%), and Russia (42%). Among 179 measles deaths reported in EUR countries during 2009–2018, 114 (64%) occurred during 2017–2018, including 93 (82%) from four countries: Romania (46), Ukraine (20), Serbia (15), and Italy (12). EUR reported 17,587 measles virus sequences to the WHO global measles nucleotide surveillance database. The most predominant measles virus genotypes detected were D4 (21% overall, 66% during 2009–2012), D8 (45% overall, 76% during 2013–2016), and B3 (33% overall, 58% during 2017–2018) (*8*) (Supplementary Figure, https://stacks.cdc.gov/view/cdc/77667).

## Regional Verification of Measles Elimination

The European Regional Verification Commission for Measles and Rubella Elimination was established in 2011 to evaluate the status of measles and rubella elimination^¶^ in EUR countries based on documentation submitted annually by national verification committees (*1*). By the end of 2017, 43 (91%) countries had interrupted endemic measles virus transmission for ≥12 months, including 37 (70%)** that had sustained interruption for ≥36 months and were verified to have eliminated endemic measles virus transmission (*8*).

## Discussion

After relatively stable albeit high measles incidence in EUR during 2009–2016, the number of reported measles cases tripled from 2017 to 2018, including outbreaks in eight countries reporting >2,000 measles cases each. The 2018 measles resurgence was attributable to measles virus transmission that began in 2017 and continued during 2018 in France, Greece, Romania, Russia, Serbia, and Ukraine. In addition, measles virus importations followed by widespread measles virus transmission occurred in countries that had achieved elimination, including Albania, Belarus, Czech Republic, Israel, and Montenegro. Despite high reported national coverage, factors associated with the resurgence included persistent measles virus reservoirs in EUR countries with limited resources and weak immunization systems, an accumulation of susceptible young children in marginalized communities with suboptimal coverage, and an accumulation of susceptible young adults who had escaped both natural measles infection and measles vaccination over a prolonged period of decreased measles incidence.

Outbreak response differed among countries. In some countries, large outbreaks caused substantial financial and human resource burdens, which resulted in delayed or inadequate outbreak responses and ongoing disease transmission. In other countries, outbreak response vaccination campaigns were not implemented because of insufficient political commitment, poor acceptance of mass immunization by health authorities and the public, lack of infrastructure to vaccinate specific susceptible population groups, and vaccine supply challenges. To achieve better outbreak control, countries in the region will need to adhere to their commitment to eliminate measles and rubella and ensure that dedicated financial and human resources are available for strong vaccination and surveillance programs, including outbreak preparedness and response.

The measles resurgence and the European Vaccine Action Plan midterm review in 2018 (*9*) highlighted ongoing challenges, including inadequate vaccine delivery infrastructure in some middle-income countries that resulted in suboptimal vaccination coverage and vaccine stock-outs; prevalent antivaccine sentiment; large populations of unvaccinated persons, including ethnic and religious minorities and adults; an increased proportion of cases in persons aged ≥20 years, who are difficult to reach with routine immunization services; and nosocomial outbreaks that affected patients and health care personnel with spread to the community.

To address these challenges and accelerate measles elimination efforts in EUR, the European Regional Office has targeted the following areas for action: 1) achieving and maintaining ≥95% vaccination coverage; 2) improving understanding of barriers to vaccination in vulnerable groups and increasing vaccine demand; 3) closing immunity gaps in the population through innovative and locally tailored approaches; 4) ensuring high-quality measles surveillance for rapid case detection and targeted outbreak response activities; and 5) strengthening infection prevention and control practices, particularly during outbreaks. The midterm review also highlighted the recent recommendation by the WHO Strategic Advisory Group of Experts on Immunization that countries institutionalize school entry checks to close immunity gaps as a key strategy for achieving measles elimination (*10*).

The findings in this report are subject to at least two limitations. First, surveillance data likely underestimate actual disease incidence because not all patients seek care, and it is likely that not all cases are reported. Second, measles surveillance performance and data quality vary among countries in the region, which might have led to reporting bias for some countries.

In EUR, 70% of countries have been verified as having achieved measles elimination; however, the recent resurgence highlighted challenges to achieving and maintaining elimination. All countries need to strengthen immunization programs to achieve and sustain high population immunity, maintain high-quality surveillance, and ensure outbreak preparedness and prompt response to contain outbreaks. Elimination efforts that focus on reaching vulnerable communities and adults will likely provide opportunities to improve access to vaccination services for all and help achieve European Vaccine Action Plan and future universal health goals.

**Summary**

**What is already known about this topic?**

Many countries in the World Health Organization European Region (EUR) have made substantial progress toward measles elimination.

**What is added by this report?**

By end of 2017, 37 (70%) EUR countries had sustained interruption of measles transmission for ≥36 months and were verified to have eliminated endemic measles. During 2017–2018, however, a resurgence of measles occurred in EUR, with large-scale outbreaks in Ukraine, Serbia, and some countries that had achieved elimination.

**What are the implications for public health practice?**

To achieve regional measles elimination, measures are needed to strengthen immunization programs to achieve high population immunity, maintain high-quality surveillance for rapid case detection, and ensure outbreak preparedness and prompt response to contain outbreaks.

## Footnotes

* The European Region, with a population of approximately 900 million, is one of six WHO regions and consists of 53 countries: Albania, Andorra, Armenia, Austria, Azerbaijan, Belarus, Belgium, Bosnia and Herzegovina, Bulgaria, Croatia, Cyprus, Czech Republic, Denmark, Estonia, Finland, France, Georgia, Germany, Greece, Hungary, Iceland, Ireland, Israel, Italy, Kazakhstan, Kyrgyzstan, Latvia, Lithuania, Luxembourg, Malta, Monaco, Montenegro, Netherlands, North Macedonia, Norway, Poland, Portugal, Republic of Moldova, Romania, Russia, San Marino, Serbia, Slovakia, Slovenia, Spain, Sweden, Switzerland, Tajikistan, Turkey, Turkmenistan, Ukraine, United Kingdom, and Uzbekistan.

† For Iceland, Norway, and the 28 member states of the European Union (Austria, Belgium, Bulgaria, Croatia, Cyprus, Czech Republic, Denmark, Estonia, Finland, France, Germany, Greece, Hungary, Ireland, Italy, Latvia, Lithuania, Luxembourg, Malta, Netherlands, Poland, Portugal, Romania, Slovakia, Slovenia, Spain, Sweden, and the United Kingdom).

§ Belgium, Bosnia and Herzegovina, Kazakhstan, North Macedonia, Serbia, and Ukraine report aggregated surveillance data to WHO.

¶ Elimination defined as interruption of endemic measles transmission for >36 months in the presence of a well-functioning surveillance system.

** Countries that had interrupted endemic measles virus transmission for >12 months include Albania, Andorra, Armenia, Azerbaijan, Belarus, Bulgaria, Croatia, Cyprus, Czech Republic, Denmark, Greece, Estonia, Finland, Hungary, Iceland, Ireland, Israel, Latvia, Lithuania, Luxembourg, Malta, Montenegro, Netherlands, North Macedonia, Norway, Monaco, Portugal, Republic of Moldova, San Marino, Slovakia, Slovenia, Spain, Sweden, Tajikistan, Turkmenistan, United Kingdom, and Uzbekistan.
